# The dark side of emotion: the addiction perspective

**DOI:** 10.1016/j.ejphar.2014.11.044

**Published:** 2015-01-09

**Authors:** George F. Koob

**Affiliations:** Director, National Institute on Alcohol Abuse and Alcoholism, Washington DC (on leave of absence from Committee on the Neurobiology of Addictive Disorders, The Scripps Research Institute, La Jolla, CA, USA)

**Keywords:** opponent process, extended amygdala, corticotropin-releasing factor, dynorphin, incentive salience, emotion, allostasis

## Abstract

Emotions are “feeling” states and classic physiological emotive responses that are interpreted based on the history of the organism and the context. Motivation is a persistent state that leads to organized activity. Both are intervening variables and intimately related and have neural representations in the brain. The present thesis is that drugs of abuse elicit powerful emotions that can be interwoven conceptually into this framework. Such emotions range from pronounced euphoria to a devastating negative emotional state that in the extreme can create a break with homeostasis and thus an allostatic hedonic state that has been considered key to the etiology and maintenance of the pathophysiology of addiction. Drug addiction can be defined as a three-stage cycle—*binge/intoxication*, *withdrawal/negative affect*, and *preoccupation/anticipation*—that involves allostatic changes in the brain reward and stress systems. Two primary sources of reinforcement, positive and negative reinforcement, have been hypothesized to play a role in this allostatic process. The negative emotional state that drives negative reinforcement is hypothesized to derive from dysregulation of key neurochemical elements involved in the brain incentive salience and stress systems. Specific neurochemical elements in these structures include not only decreases in incentive salience system function in the ventral striatum (within-system opponent processes) but also recruitment of the brain stress systems mediated by corticotropin-releasing factor (CRF), dynorphin-κ opioid systems, and norepinephrine, vasopressin, hypocretin, and substance P in the extended amygdala (between-system opponent processes). Neuropeptide Y, a powerful anti-stress neurotransmitter, has a profile of action on compulsive-like responding for drugs similar to a CRF_1_ antagonist. Other stress buffers include nociceptin and endocannabinoids, which may also work through interactions with the extended amygdala. The thesis argued here is that the brain has specific neurochemical neurocircuitry coded by the hedonic extremes of pleasant and unpleasant emotions that have been identified through the study of opponent processes in the domain of addiction. These neurochemical systems need to be considered in the context of the framework that emotions involve the specific brain regions now identified as differentially interpreting emotive physiological expression.

## 1. What is Emotion?

Emotion can be defined as “a psychic and physical reaction (as anger or fear) subjectively experienced as strong feeling and physiologically involving changes that prepare the body for immediate vigorous action” (Webster’s Ninth New Collegiate Dictionary, 1984). The introspective emphasis on the “feeling” aspect of emotions had a prominent role in the development of many theories of emotion. Darwin argued as early as 1872 that both observable expressions of emotions as well as underlying brain processes (direct action of the excited nervous system on the body) are not unique to humans (Darwin, 1872). Indeed, key emotional expressions were considered innate, instinctive responses but subject to the evolutionary process.

### 1.1. Emotional behavior vs. feelings of emotion

Emotional behavior, or the measurement of bodily changes associated with emotional behavior, focused on peripheral response mechanisms largely related to the autonomic and endocrine systems. Peripheral measures of emotion ranged from galvanic skin responses to heart rate to salivary secretion to levels of autonomic hormones. Such peripheral responses have long been difficult to separate from the feelings of emotion. Indeed, William James in his famous theory of emotion argued, “Bodily changes follow directly the *perception* of the exciting fact, and that our feeling of the same changes as they occur in *is* the emotion” (James, 1884, p. 189–190).

The brain became a key mediator of emotion by parallel advances in conceptual framework and neuroanatomical studies. Ferrier (1875) showed that orbitofrontal ablations in monkeys had no major effect on an organism’s sensory abilities but produced a definite change in the disposition of the animal. Broca (1878) described the “grand lobe limbique” (“limbic” indicates that this lobe surrounds the brain stem) which included the olfactory tubercle, prepyriform cortex, diagonal band of Broca, septal region, hippocampus, and cingulate as a common emotional circuit in all mammals. The demonstration of decorticate “sham rage” in the 1920s led to the hypothesis that emotional expression involved specific subcortical structures. Later stimulation studies by pointed to subcortical structures, such as the hypothalamus, soon to be labeled “limbic” structures in the neural circuitry of the expression of emotional responses (Masserman, 1941).

From a conceptual perspective, Cannon argued against the James-Lange Theory, largely on the basis of the observation that animals continued to express emotional behavior in the absence of information from the periphery. Later, he hypothesized that emotional experience and emotional behavior were a release from cortical inhibition of neural impulses originating in the thalamus (Cannon, 1927). Bard removed the neocortex of cats, leaving the rhinencephalon intact, which produced placidity (Bard and Mountcastle, 1948). This placidity could be changed to ferocity by removing the amygdaloid complex (Bard and Match, 1951). Bard’s extensive work made modifying Cannon’s theory possible so that it could better define the neurocircuitry of emotional behavior and led Papez to argue that the hypothalamus was critical for the expression of emotional behavior.

The Papez circuit was proposed in 1937 as a circuit for emotion and evolved into the terminology and conceptual framework of the limbic system which remains today (Papez, 1937, 1939). The Papez circuit included the cortex, cingulate gyrus, mammillary bodies, anterior thalamus, subthalamic areas, and hypothalamus. Thus, the limbic system came to represent not only Broca’s 1878 grand lobe limbique but also most allocortical regions of the brain from the Papez circuit for the subjective experience of emotion and the hypothalamus for emotional expression. MacLean later added the hippocampus and its association with the amygdala as a key part of the experience of emotion (MacLean, 1949). To some extent, the term “limbic system” has been abrogated to include any brain structure involved in emotional function, leading to a somewhat circular argument of what constitutes the limbic system.

### 1.2. Recent perspectives on the neurobiological bases of emotion

Important to our conceptual understanding of the neuroscience of emotion was the suggestion of Schachter and Singer (1962) that cognitive factors may be major determinants of emotional states. More specifically, these authors argued that cognition arising from the immediate emotional experience, as interpreted by past experience, provides the framework for labeling one’s feelings, and thus cognition determines whether a state of physiological arousal will be labeled as a given emotion (Schachter, 1975).

Later, a universality of six emotions was proposed based on extensive cross-cultural work on facial expression—happiness, surprise, fear, sadness, anger, and disgust combined with contempt—with distinctive patterns of central nervous system activity (Ekman and Friesen, 1986). Similar emotional states were hypothesized even for rodents, including distress, anger, social bonding, play, and laughter (Panksepp, 1998). Yet others, such as Russell (2003), avoided a specific categorization of emotion and argued that any emotionally charged event is a state experienced as simply feeling good or bad, energized or enervated—in other words, a free-floating mood or core affect that is subject to interpretation by the perception of affective quality.

In an integration of modern thinking with some aspects of the original James-Lange Theory, the somatic marker hypothesis of Damasio (1996) argued that decision-making is a process that is influenced by somatic (of the body, not just the muscles) marker signals that arise in bioregulatory processes, including those that express themselves in emotions and feelings. A key part of the theory is that the ventromedial cortex provides the substrate for “learning an association between certain classes of complex situation, on the one hand, and the type of bioregulatory state (including emotional state) usually associated with that class of situation in past individual experience” (Bechara et al., 2000, p. 296). From this perspective, the amygdala has been shown to be a structure that is necessary for emotions to improve memory (Cahill et al., 1995) and the creation of biases and decision making (Bechara et al., 1999).

Modern brain imaging studies have consolidated such integrative views of emotions. Morris and colleagues (1996) showed that the amygdala in humans responds differentially in subjects shown facial expressions of fear and happiness, with the neuronal response in the left amygdala significantly greater in response to fearful *vs*. happy faces. Damasio (2002), in a series of studies, argued that the term “emotion” should be defined as specific and consistent collections of physiological responses triggered by certain brain regions when the organism is presented with a specific situation. The substrates for the representation of emotions include homeostatic circuitry in the brainstem, hypothalamus, basal forebrain, amygdala, ventromedial prefrontal cortex, and cingulate cortex. In contrast, Damasio defined “feelings” as the mental states that arise from the neural representation of the collection of responses that constitute an emotion and as such should be reserved for the private, mental experience of an emotion. Key structures involved in feelings, he argued, include the brainstem, hypothalamus, thalamus, cingulate, somatosensory cortices of the insula, and somatosensory I and II. He hypothesized that to monitor cognitive processing, the prefrontal cortex is engaged. This approach led to arguments in which specific brain systems, including the posteromedial cortices (precuneus, posterior cingulate cortex, and retrosplenial region) and anterior insula, are recruited in addition to the basic homeostatic circuitry for specific types of emotions, such as social emotions (e.g., admiration and compassion; Moll et al., 2005) and for engagement of the salience system (Seeley et al., 2007; Immordino-Yang et al., 2009).

## 2. Interface between Emotion and Motivation

### 2.1. Motivation

Motivation, similar to emotion, is a concept that has many definitions, but even early definitions reflected internal elements that drive behavior. Motivation was defined as “an inner psychological process or function, a driving force to be found chiefly within the organism itself and a plan, purpose or ideal with the definite implication of an ideational element” that may not be consciously or overtly recognized (Perrin, 1923). Richter argued that “spontaneous activity arises from certain underlying physiological origins and such ‘internal’ drives are reflected in the amount of general activity” (Richter, 1927). Hebb stated that motivation is “stimulation that arouses activity of a particular kind” (Hebb, 1949). Bindra defined motivation as a “rough label for the relatively persisting states that make an animal initiate and maintain actions leading to particular outcomes or goals” (Bindra, 1976). A more behavioristic view is that motivation is “the property of energizing behavior that is proportional to the amount and quality of the reinforcer” (Kling and Riggs, 1971). Finally, a more neurobehavioral view is that motivation is a “set of neural processes that promote actions in relation to a particular class of environmental objects” (Bindra, 1976).

An early and influential theory of motivation by Hull (1943), termed the “drive-reduction theory,” was predicated on the hypothesis of homeostatic mechanisms of motivation, in which behavior could be regarded as an outward expression of the organism’s pursuit of biological equilibrium. Here, all motivation was theorized to derive from biological imbalances or needs. A “need” in this formulation, such as hunger (here, the need is for more energy), was a biological requirement of the organism. Motivation, according to Hull, then aimed at making up for or erasing a deficiency or lack of something in the organism. The word “drive” was used to describe the state of behavioral arousal resulting from a biological need and was the energy that powered behavior. Drive reduction theory fell out of favor because researchers noted that much motivated behavior could be generated without any biological drives being manifest.

### 2.2. Behavioral analysis of emotion: bridge to motivation

The role of emotion in motivation can be traced to two major sources, the experimental analysis of behavior and the neurobiological bases of brain-stimulated reward. The behavioral analysis of motivational and emotional interactions championed by Brady (1978) led to a response-inferred foundation for a unifying operational framework relating motivational and emotional function. Brady argued elegantly that the “hedonic” characteristics of motivational functions can be accommodated by appealing to the experimentally based distinctions between “positive” and “negative” reinforcement operations. The evident byproducts (e.g., “euphoria”) of “appetitive” contingencies increase the likelihood of behavior, and byproducts of the “dysphoric” accompaniments of “aversive” contingencies weaken behavior (or strengthen escape and avoidance performances; Brady and Emurian, 1978).

Under this formulation, the analysis of emotional function focuses on procedures that affect the efficacy of reinforcers in the contingency (e.g., food deprivation potentiates the consequence or increases the likelihood of consummatory behaviors, or startle effects may disrupt the efficacy of reinforcers and decrease the likelihood of a consummatory behavior). Such emotional functions emphasize the prominent role of inner events (e.g., feelings), and as such terms that are related to hedonic function could be accommodated in terms of positive reinforcement, negative reinforcement, and punishment. Thus, the hedonic dimension of Brady’s “behavioral universe” refers primarily to the affective valence of the bridge between emotion and motivation. Indeed, emotions are often linked to motivation but are not necessary for motivation.

All of these conceptualizations led to the development of incentive motivation theory largely led by Bolles (Bolles, 1975) and followed by the work of Bindra (1974) and Toates (1981). Bolles argued that many issues remained unexplained by drive reduction theory, but a major observation was that individuals were motivated by incentive expectancies or learned expectancies of reward or what in Pavlovian associations are conditioned and unconditioned stimuli (CS and US). Bindra argued that the CS for a reward came to elicit the same motivational state as the reward itself, thus causing the individual to perceive the CS as a reward, not just the expectancy of reward. Toates (1981) further refined the model by arguing that physiological states (drive states) could enhance the incentive value of the reward. Modern incentive-motivation theory, which has guided the neurobiology of motivation, has many contributions that range from two-process learning theory to instrumental incentive learning (Rescorla and Solomon, 1967; Dickinson and Balleine, 2002).

The concept of incentive motivation was bolstered by the discovery that electrical stimulation of the medial forebrain bundle was highly rewarding to animals, providing a key experimental bridge of the neurobiology of emotion with the neurobiology of motivation (Olds and Milner,1954). Brain stimulation reward involves widespread neurocircuitry throughout the brain, but the most sensitive sites include the trajectory of the medial forebrain bundle that connects the ventral tegmental area with the basal forebrain (Olds and Milner, 1954; Koob et al., 1977; Simon et al., 1979). Early on, theorists such as J. Anthony Deutsch, argued had that brain stimulation reward activated two pathways: a pathway conveying reinforcement and a pathway conveying excitation (motivation). This was another early link between emotion (hedonic function) and motivation (Deutsch, 1960). Later work showed that brain stimulation reward had elements of both motivation and reinforcement, and behavior mediated by brain stimulation reward resembled other high-incentive-maintained behavior that was delivered quickly and did not require a “drive “ state *per se* (Panksepp and Trowill, 1967a, b). Perhaps more to the point, when brain stimulation was delivered as a bout of nosepoking behavior, it sustained responding on intermittent schedules of reinforcement, similar to food in food-deprived animals (Pliskoff et al., 1965).

Contributing to the motivational construct of incentive motivation was a subsequent observation that animals and humans would readily intravenously self-administer drugs that in humans have positive hedonic effects. No drive state needed to be established (Panlilio and Goldberg, 2007). All drugs that are self-administered in humans also decrease brain stimulation reward thresholds (i.e., increase or facilitate reward; Kornetsky and Esposito, 1979). Although much emphasis was initially placed on the role of ascending monoamine systems as the mediating neurochemical systems, particularly the dopamine system, in the medial forebrain bundle, other non-dopaminergic systems in the medial forebrain bundle clearly play a key role (Hernandez et al., 2006; Garris et al., 1999; Miliaressis et al., 1991).

## 3. Incentive Salience

Subsequent theories of motivation have linked motivation with hedonic, affective, or emotional states and postulated changes in motivation over time and experience.

Drugs that have high reinforcing value also have a profound effect on previously neutral stimuli to which they have been paired, termed a facilitation of “incentive salience.” Originally, incentive salience was described as incentive attribution. Here, “if an associative representation is to alter the course of subsequent behavior, then that action and its eliciting stimulus or representation must actively be attributed with incentive value to elicit approach and to initiate instrumental behavior on future occasions” (Berridge and Valenstein, 1991, p. 10). Incentive salience attribution was hypothesized to be linked directly to activation of the mesocorticolimbic dopamine system (Berridge and Valenstein, 1991).

Early work in behavioral pharmacology on stimulants showed that these drugs could facilitate “conditioned reinforcement.” Given the role of dopamine in mediating the actions of psychostimulants, this provides strength to the incentive salience hypothesis from another theoretical perspective. Here, psychostimulants would cause rats to show compulsive-like lever pressing for the cue that had been paired with a water reward (Robbins, 1976). Later, a neurobiological substrate for such conditioned reinforcement/incentive salience was provided in a series of studies that recorded from dopamine neurons in the ventral tegmental area in primates during repeated presentation of rewards and presentation of stimuli associated with reward. Dopamine cells fired upon the first exposure to a novel reward, but repeated exposure to dopamine caused the neurons to stop firing upon reward consumption and fire instead when they were exposed to stimuli that were *predictive* of the reward (Schultz et al., 1997). This suggested a role for phasic dopamine cell firing, which leads to large and transient dopamine activity, in conditioned reinforcement, which later was translated to incentive salience via the conceptual framework of incentive sensitization.

The incentive-sensitization (or incentive salience) theory of Robinson and Berridge (1993) divided, but in a sense extended, the power of incentives and moved them to a neuroadaptive trajectory. Here, incentives were split into two components—“wanting” and “liking”—based on the hypothesis that different brain mechanisms mediate these separate components. “Liking” was hypothesized to be mediated by opioid systems in the nucleus accumbens and ventral pallidum and defined as the hedonic impact or brain response to sensory reward or pleasure without motivational power. In contrast, “wanting” was hypothesized to be mediated by the mesocorticolimbic dopamine system and argued to be the incentive salience or motivational incentive value of the same reward. Little independent evidence has been generated to separate “wanting” from “liking,” but the construct of incentive salience has gained significant traction.

As noted previously, all drugs abuse can initially elicit the supra-physiological phasic release of dopamine in the nucleus accumbens. This drug-induced phasic dopamine signaling can eventually trigger neuroadaptations in other basal ganglia circuits that are related to habit formation. Key synaptic changes involve the recruitment of glutamate-modulated *N*-methyl-D-aspartate (NMDA) and α-amino-3-hydroxy-5-methyl-4-isoxazolepropionic acid receptors in glutamatergic projections from the prefrontal cortex and amygdala to the ventral tegmental area and nucleus accumbens (Kalivas, 2009; Luscher and Malenka, 2011; Pierce and Wolf, 2013). The power of initial dopamine release (and activation of opioid peptide systems) upon initial drug taking begins the neuroadaptations that lead to tolerance and withdrawal and triggers drug-associated cue exposure to raise dopamine levels in the dorsal striatum, a region with a key role in habit formation (Belin et al., 2013), likely supporting the strengthening of habits (even those uncoupled from actual drug delivery) as addiction progresses. The subsequent recruitment of cortical-striatal-pallidal-thalamic circuits is significant for progression through the addiction cycle because such conditioned responses help explain the intense desire for the drug (craving) and compulsive use when subjects with addiction are exposed to drug cues. Thus, conditioned responses in the incentive salience process may drive dopamine signaling that maintains the motivation to take the drug, even when its pharmacological effects appear attenuated.

In summary, the observations mentioned above trace the history of motivation and point to certain common characteristics of our concept of motivation. Motivation is a state that varies with arousal and guides behavior in relationship to changes in the environment. The environment can be external (incentives) or internal (central motive states or drives), and such motivation or motivational states are not constants but rather vary over time. A classic example of motivational states that profoundly change over time is the pathophysiology we term addiction.

## 4. Addiction as a Model of Emotional Dysregulation

### 4.1. What is addiction?

Addiction can be defined as a chronic, relapsing disorder that has been characterized by (*i*) a compulsion to seek and take drugs, (*ii*) loss of control over drug intake, and (*iii*) emergence of a negative emotional state (e.g., dysphoria, anxiety, and irritability) that defines a motivational withdrawal syndrome when access to the drug is prevented (Koob and Le Moal, 1997). The occasional, limited, recreational use of a drug is clinically distinct from escalated drug use, the loss of control over drug intake, and the emergence of compulsive drug-seeking behavior that characterize addiction.

Addiction has been conceptualized as a three-stage cycle—*binge/intoxication*, *withdrawal/negative affect*, and *preoccupation/anticipation*—that worsens over time and involves allostatic changes in the brain reward and stress systems. Two primary sources of reinforcement, positive and negative reinforcement, have been hypothesized to play a role in this allostatic process (see above discussion of the seminal work of Brady). Positive reinforcement is defined as the process by which presentation of a stimulus increases the probability of a response; negative reinforcement is defined as the process by which removal of an aversive stimulus (or aversive state in the case of addiction) increases the probability of a response.

### 4.2. Neurobiology of addiction

The neurobiological basis of the *binge/intoxication* stage involves the facilitation of incentive salience (see above) and is mediated largely by neurocircuitry in the basal ganglia. The basal ganglia are associated with a number of key functions, including voluntary motor control, procedural learning related to routine behaviors or habits, and action selection. The release of dopamine and opioid peptides in the ventral striatum (nucleus accumbens) has long been associated with the reinforcing actions of drugs of abuse. As a result, drugs of abuse confer motivational properties to previously neutral stimuli, known as incentive salience. For example, human imaging studies have shown that intoxicating doses of most drugs of abuse and alcohol release dopamine and opioid peptides into the ventral striatum (Volkow et al., 2007; Mitchell et al., 2012). The activation of the ventral striatum leads to the recruitment of basal ganglia-globus pallidus-thalamic-cortical loops that engage the dorsal striatum habit formation and strengthening hypothesized to be the beginning of compulsive-like responding for drugs (see above).

Subsequently and perhaps in parallel, two processes are hypothesized to form the neurobiological basis for the *withdrawal/negative affect* stage: loss of function in the reward systems (within-system neuroadaptation) in the ventral striatum and recruitment of the brain stress systems (between-system neuroadaptation) in the extended amygdala. The extended amygdala has been conceptualized to be composed of several basal forebrain structures, including the bed nucleus of the stria terminalis, central nucleus of the amygdala, sublenticular substantia innominata, and a transition zone in the medial part of the nucleus accumbens (e.g., shell; Heimer and Alheid, 1991). As dependence (defined as the manifestation of motivational withdrawal symptoms) develops, brain stress systems, such as CRF, norepinephrine, dynorphin, hypocretin, and substance P, are recruited, producing aversive or stress-like states (Koob and Le Moal, 2001; Carlezon et al., 2000). The combination of decreases in reward neurotransmitter function and recruitment of brain stress systems provides a powerful motivation for reengaging in drug taking and drug seeking.

The *preoccupation/anticipation* stage in addiction mediates the dysregulation of executive control via prefrontal cortex circuits. The global function of the prefrontal cortex is to engage executive function. To accomplish such complex tasks in the domain of addiction, one can conceptualize two opposing systems, a Go system and a Stop system. The Go system consists of the anterior cingulate cortex and dorsolateral prefrontal cortex and engages habits via the basal ganglia. The Stop system consists of the ventral prefrontal cortex and orbitofrontal cortex and inhibits the basal ganglia incentive salience system and the extended amygdala stress system. In individuals with substance use disorders, there are disruptions of decision making, impairments in the maintenance of spatial information, and impairments in behavioral inhibition, all of which can drive craving. Craving, defined as the desire for the drug or alcohol in the absence of the drug, can also be divided into two domains: reward craving (drug seeking induced by drugs or stimuli linked to drugs) and relief craving (drug seeking induced by an acute stressor or a state of stress. Both of these constructs parallel the hypothesized subcortical dysregulations associated with the *binge/intoxication* and *withdrawal negative-affect* stages and can contribute to relapse.

## 5. Dynamic Changes in Reward: Allostatic View of Opponent Processes

**Fig. 1:**
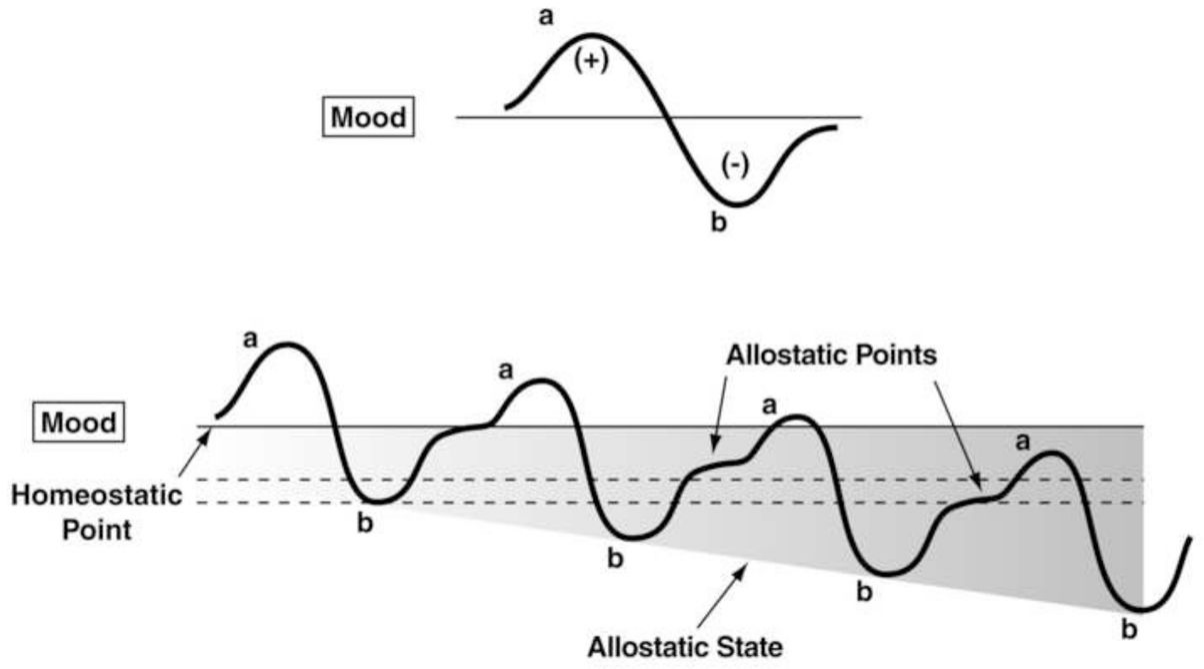
Diagram illustrating an extension of Solomon and Corbit’s (1974) opponent-process model of motivation to incorporate the conceptual framework of this paper. All panels represent the affective response to the presentation of a drug. **(Top)** This diagram represents the initial experience of a drug with no prior drug history, and the a-process represents a positive hedonic or positive mood state and the b-process represents the negative hedonic of negative mood state. The affective stimulus (state) has been argued to be a sum of both an a-process and a b-process. An individual whom experiences a positive hedonic mood state from a drug of abuse with sufficient time between re-administering the drug is hypothesized to retain the a-process. In other words, an appropriate counteradaptive opponent-process (b-process) that balances the activational process (a-process) does not lead to an allostatic state. **(Bottom)** The changes in the affective stimulus (state) in an individual with repeated frequent drug use that may represent a transition to an allostatic state in the brain reward systems and, by extrapolation, a transition to addiction (see text). Note that the apparent b-process never returns to the original homeostatic level before drug-taking begins again, thus creating a greater and greater allostatic state in the brain reward system. In other words, here the counteradaptive opponent-process (b-process) does not balance the activational process (a-process) but in fact shows a residual hysteresis. While these changes are exaggerated and condensed over time in the present conceptualization, the hypothesis here is that even during post-detoxification, a period of “protracted abstinence,” the reward system is still bearing allostatic changes (see text). The following definitions apply: *allostasis*, the process of achieving stability through change; *allostatic state*, a state of chronic deviation of the regulatory system from its normal (homeostatic) operating level; *allostatic load*, the cost to the brain and body of the deviation, accumulating over time, and reflecting in many cases pathological states and accumulation of damage. [Taken with permission from Koob and Le Moal, 2001]

Changes in reinforcement were inextricably linked with hedonic, affective, or emotional states in addiction in the context of temporal dynamics by Solomon’s opponent process theory of motivation. Solomon and Corbit (1974) postulated that hedonic, affective, or emotional states, once initiated, are automatically modulated by the central nervous system through mechanisms that reduce the intensity of hedonic feelings. The *a-process* includes affective or hedonic habituation (or tolerance), and the *b-process* includes affective or hedonic withdrawal (abstinence). The thesis we elaborated previously is that there is a neurocircuitry change in specific neurochemical systems that account for the *b-process* (Koob and Le Moal, 2001). Such opponent processes were hypothesized to begin early in drug taking, reflecting not only deficits in brain reward system function but also the recruitment of brain stress systems. Furthermore, we hypothesized that the recruitment of brain stress systems forms one of the major sources of negative reinforcement in addiction. Finally, we hypothesized that such changes result not in a return to homeostasis of reward/stress function but allostasis-like dysfunction of reward/stress function that continues to drive the addiction process (Fig. 1).

Allostasis, originally conceptualized to explain the persistent morbidity of arousal and autonomic function, can be defined as “stability through change.” Allostasis involves a feed-forward mechanism rather than the negative feedback mechanisms of homeostasis, with continuous reevaluation of need and continuous readjustment of all parameters toward new set points. An *allostatic state* was defined as a state of chronic deviation of the regulatory system from its normal (homeostatic) operating level (Koob and Le Moal, 2001). *Allostatic load* was defined as the “long-term cost of allostasis that accumulates over time and reflects the accumulation of damage that can lead to pathological states” (McEwen, 2000).

Opponent process-like negative emotional states have been characterized in humans by acute and protracted abstinence from all major drugs of abuse (American Psychiatric Association, 1994; Koob, 2013; Khantzian, 1997). Similar results have been observed in animal models with all major drugs of abuse using intracranial self-stimulation (ICSS) as a measure of hedonic tone. Spontaneous or precipitated withdrawal from chronic cocaine (Markou and Koob, 1991), amphetamine (Paterson et al., 2000), opioids (Schulteis et al., 1994), cannabinoids (Gardner and Vorel, 1998), nicotine (Epping-Jordan et al., 1998), and ethanol (Schulteis et al., 1995) leads to increases in reward threshold during acute abstinence, and some of these elevations in thresholds can last for up to 1 week (Koob, 2009). Such elevations in reward threshold begin rapidly and can be observed within a single session of self-administration (Kenny et al., 2003), bearing a striking resemblance to human subjective reports of acute withdrawal. Dysphoria-like responses also accompany acute opioid and ethanol withdrawal (Liu and Schulteis, 2004; Schulteis and Liu, 2006). Here, naloxone administration following single injections of morphine increased reward thresholds, measured by ICSS, and increased thresholds with repeated morphine and naloxone-induced withdrawal experience (Liu and Schulteis, 2004). Similar results were observed during repeated acute withdrawal from ethanol (Schulteis and Liu, 2006).

### 5.1. Animals escalate their intake of drugs with extended access, with a parallel increase in reward thresholds

The hypothesis that compulsive drug use is accompanied by a chronic perturbation in brain reward homeostasis has been tested in animal models of escalation in drug intake with prolonged access combined with measures of brain stimulation reward thresholds. Animals implanted with intravenous catheters and allowed differential access to intravenous self-administration of cocaine showed increases in cocaine self-administration from day to day in the long-access group (6 h; LgA) but not in the short-access group (1 h; ShA). The differential exposure to cocaine self-administration had dramatic effects on reward thresholds that progressively increased in LgA rats but not ShA or control rats across successive self-administration sessions (Ahmed et al., 2002). Elevations in baseline reward thresholds temporally preceded and were highly correlated with escalation in cocaine intake (Ahmed et al., 2002). Post-session elevations in reward thresholds failed to return to baseline levels before the onset of each subsequent self-administration session, thereby deviating more and more from control levels. The progressive elevation in reward thresholds was associated with a dramatic escalation in cocaine consumption that was observed previously (Ahmed et al., 2002). Similar results have been observed with extended access to methamphetamine (Jang et al., 2013) and heroin (Kenny et al., 2006) and nicotine (Harris et al., 2011). These observations lend credence to the hypothesis that opponent processes in the hedonic domain have an identifiable neurobiological basis and provide an impetus for defining the mechanisms involved.

## 6. Neuroadaptations Responsible for Opponent Processes

One hypothesis is that drug addiction progresses from a source of positive reinforcement that may indeed involve a form of sensitization of incentive salience, as argued by Robinson and Berridge (1993). Little evidence has been obtained from neurobiological or clinical studies to support this hypothesis.

A second hypothesis is that drug addiction progresses via the sensitization of opponent processes that set up a powerful negative reinforcement process. A further elaboration of the second hypothesis is that there are both within- and between-system neuroadaptations to excessive activation of the reward system at the neurocircuitry level. Within-system neuroadaptations are defined as the process by which the primary cellular response element to the drug (circuit A) itself adapts to neutralize the drug’s effects. Persistence of the opposing effects after the drug disappears produces adaptation. A between-system neuroadaptation is a circuitry change, in which B circuits (i.e., the stress or anti-reward circuits) are activated by circuit A (i.e., the reward circuit). In the present treatise, within-system neuroadaptations can dynamically interact with a between-system neuroadaptation, in which circuit B (i.e., the anti-reward circuit) is activated either in parallel or in series to suppress the activity of circuit A.

## 7. Brain Reward System Substrates for the Negative Reinforcement Associated with Addiction (Within-System Neuroadaptations)

As noted above, the *withdrawal/negative affect* stage can be defined as the presence of motivational signs of withdrawal in humans, including chronic irritability, physical pain, emotional pain (i.e., hyperkatifeia; Shurman et al., 2010), malaise, dysphoria, alexithymia, and loss of motivation for natural rewards. Neurochemical mechanisms for such effects in the within domain include common effects, such as decreases in dopaminergic transmission in the ventral striatum (nucleus accumbens) during drug withdrawal measured by *in vivo* microdialysis (Parsons and Justice, 1993; Weiss et al., 1992). However, there are also drug-specific changes, such as increased sensitivity of opioid receptor transduction mechanisms in the nucleus accumbens during opioid withdrawal (Stinus et al., 1990), decreased GABAergic and increased *N*-methyl-D-aspartate (NMDA) glutamatergic transmission during alcohol withdrawal (Davidson et al., 1995; Morrisett, 1994; Roberts et al., 1996; Weiss et al., 1996), and differential regional changes in nicotinic receptor function (Collins et al., 1990; Dani and Heinemann, 1996).

Human imaging studies of individuals with addiction during withdrawal or protracted abstinence have generated results that are consistent with animal studies. There are decreases in dopamine D_2_ receptors (hypothesized to reflect hypodopaminergic functioning), hyporesponsiveness to dopamine challenge (Volkow et al., 2003), and hypoactivity of the orbitofrontal-infralimbic cortex system (Volkow et al., 2003). These are hypothesized to be within-system neuroadaptations that may reflect presynaptic release and/or postsynaptic receptor plasticity.

In the context of chronic drug administration, multiple molecular mechanisms have been hypothesized to be engaged by the repeated activation of incentive salience/reward systems that could be considered within-system neuroadaptations. Such within system molecular changes include the perturbation of intracellular signal transduction pathways. Paralleling diminished dopamine function in the nucleus accumbens, chronic administration of cocaine, methamphetamine, and heroin reduces the inhibitory G-protein subunits G_iα_ and G_oα_ in the nucleus accumbens. A reduction of the inhibitory G-protein subunits G_iα_ and G_oα_ increases cocaine self-administration in animal models and shifts the cocaine dose-response function upward with repeated sessions, identical to the effects observed with extended access to intravenous cocaine self-administration (Self et al., 1994), an effect also produced by injections of pertussis toxin injected into the nucleus accumbens (Edwards and Koob, 2010). Paralleling the reductions of inhibitory G-protein levels, chronic exposure to a wide variety of abused drugs upregulates cyclic adenosine monophosphate (cAMP) formation, cAMP-dependent protein kinase A (PKA) activity, and PKA-dependent protein phosphorylation in the nucleus accumbens, all of which can produce escalated drug self-administration or enhanced drug-seeking behavior. Thus, upregulation of a postsynaptic G_s_/cAMP/PKA signaling pathway in the nucleus accumbens may constitute a critical neuroadaptation that is central to the establishment and maintenance of the addicted state (Edwards and Koob, 2010) and parallel the increased motivation that one observes in animal models of compulsive drug seeking that are characterized by negative emotional states during withdrawal.

In turn, such changes in signal transduction can trigger longer-term molecular neuroadaptations via transcription factors that can modify gene expression and consequently protein formation. Two transcription factors in particular have been implicated in the plasticity associated with addiction: cAMP response element binding protein (CREB) and ΔFosB. CREB can be phosphorylated by PKA and protein kinases that are regulated by growth factors, putting it at a point of convergence for several intracellular messenger pathways that can regulate the expression of genes. Much work in the addiction field has shown that CREB activation in the nucleus accumbens follows chronic exposure to opiates, cocaine, and alcohol and that CREB deactivation in the central nucleus of the amygdala follows chronic exposure to alcohol and nicotine. As noted above, the activation of CREB in the nucleus accumbens with psychostimulant and opioid drugs is linked to the motivational symptoms of withdrawal, possibly through induction of the opioid peptide dynorphin. More specifically, these molecular adaptations may decrease an individual’s sensitivity to the rewarding effects of subsequent drug exposure (tolerance) and decrease reward pathway function so that after removal of the drug the individual is left in an amotivational, dysphoric, or depressed-like state (withdrawal; Nestler, 2004). In contrast, decreased CREB phosphorylation has been observed in the central nucleus of the amygdala during alcohol withdrawal and has been linked to decreased NPY function and consequently the increased anxiety-like responses associated with acute alcohol (Moonat et al., 2010). Importantly, increased CREB in the nucleus accumbens and decreased CREB in the central nucleus of the amygdala are not necessarily mutually exclusive and point to transduction mechanisms that can produce neurochemical changes in the neurocircuits outlined above to induce breaks with reward–stress homeostasis in addiction.

Transcription factors such as CREB can also change gene expression and produce long-term changes in protein expression and, as a result, neuronal function (Nestler, 2005). One group of transcription factors, isoforms of ΔFosB, accumulate over longer periods of time (over days) with repeated drug administration (Nestler, 2004). Animals with activated ΔFosB exhibit exaggerated sensitivity to the rewarding effects of drugs of abuse. Nestler has argued that ΔFosB may be a sustained molecular trigger or a sustained molecular “switch” that helps to initiate and maintain a state of addiction (Nestler, 2005). Such a molecular framework has led to the discovery of the activation of other transcription factors, such as nuclear factor κB and CDK5, which can trigger even structural changes in the cytoskeleton of neurons via actions on actin (Russo et al., 2010). Thus, within-system molecular changes may form a critical juncture for genetic epigenetic variability in the vulnerability to negative emotional states.

## 8. Brain Stress System Substrates for the Negative Reinforcement Associated with Addiction (Between-System Neuroadaptations)

Brain neurochemical systems involved in arousal-stress modulation have been hypothesized to be engaged within the neurocircuitry of the brain stress systems in an attempt to overcome the chronic presence of the perturbing drug and restore normal function despite the presence of drug (Koob, 2008). Both the hypothalamic-pituitary-adrenal (HPA) axis and extrahypothalamic brain stress system mediated by CRF are dysregulated by chronic administration of all major drugs with dependence or abuse potential, with a common response of elevated adrenocorticotropic hormone, corticosterone, and amygdala CRF during acute withdrawal (Rivier et al., 1984; Merlo-Pich et al., 1995; Koob et al., 1994; Rasmussen et al., 2000; Olive et al., 2002; Delfs et al., 2000; Koob, 2009; Roberto et al., 2010). Indeed, activation of the HPA response may be an early dysregulation associated with excessive drug taking that ultimately “sensitizes” the extrahypothalamic CRF systems (Koob and Kreek, 2007; Vendruscolo et al., 2012).

In parallel with the activation of the stress response system is the manifestation of anxiety-like responses that are reversed by CRF antagonists during acute withdrawal and protracted abstinence from all major drugs of abuse. Withdrawal from repeated administration of cocaine produces an anxiogenic-like response in the elevated plus maze and defensive burying test, both of which are reversed by administration of CRF receptor antagonists (Sarnyai et al., 1995; Basso et al., 1999). Opioid dependence also produces irritability-like effects that are reversed by CRF receptor antagonists (Navarro-Zaragosa et al., 2010; Iredale et al., 2000). Ethanol withdrawal produces anxiety-like behavior that is reversed by intracerebroventricular administration of CRF_1_/CRF_2_ peptidergic antagonists (Baldwin et al., 1991) and small-molecule CRF_1_ antagonists (Knapp et al., 2004; Overstreet et al., 2004; Funk et al., 2007) and intracerebral administration of a peptidergic CRF_1_/CRF_2_ antagonist into the amygdala (Rassnick et al., 1993). The effects of CRF antagonists have been localized to the CeA (Rassnick et al., 1993). Precipitated withdrawal from nicotine produces anxiety-like responses that are also reversed by CRF antagonists (Tucci et al., 2003; George et al., 2007). CRF antagonists injected intracerebroventricularly or systemically also block the potentiated anxiety-like responses to stressors observed during protracted abstinence from chronic ethanol (Breese et al., 2005; Valdez et al., 2003; Huang et al., 2010; Overstreet et al., 2007; Wills et al., 2009).

Another measure of negative emotional states during drug withdrawal in animals is conditioned place aversion, in which animals avoid an environment previously paired with an aversive state. Such place aversions, when used to measure the aversive stimulus effects of withdrawal, have been observed largely in the context of opioids (Hand et al., 1988; Stinus et al., 1990). Systemic administration of a CRF_1_ receptor antagonist and direct intracerebral administration of a peptide CRF_1_/CRF_2_ antagonist also decreased opioid withdrawal-induced place aversions (Stinus et al., 2005; Heinrichs et al., 1995; Contarino and Papaleo, 2005). These effects have been hypothesized to be mediated by actions in the extended amygdala. The selective CRF_1_ antagonist antalarmin blocked the place aversion produced by naloxone in morphine-dependent rats (Stinus et al., 2005), and a CRF peptide antagonist injected into the CeA also reversed the place aversion produced by methylnaloxonium injected into the CeA (Heinrichs et al., 1995). CRF_1_ knockout mice failed to show conditioned place aversion to opioid withdrawal and failed to show an opioid-induced increase in dynorphin mRNA in the nucleus accumbens (Contarino and Papaleo, 2005).

A compelling test of the hypothesis that CRF-induced increases in anxiety-like responses during drug withdrawal has motivational significance in contributing to negative emotional states is the observation that CRF antagonists can reverse the elevation in reward thresholds produced by drug withdrawal. Nicotine and alcohol withdrawal-induced elevations in reward thresholds were reversed by a CRF antagonist (Bruijnzeel et al., 2007, 2010). These effects have been localized to both the central nucleus of the amygdala and nucleus accumbens shell (Marcinkiewcz et al., 2009).

### 8.1. Corticotropin-releasing factor, compulsive-like drug seeking, and the extended amygdala

**Fig. 2:**
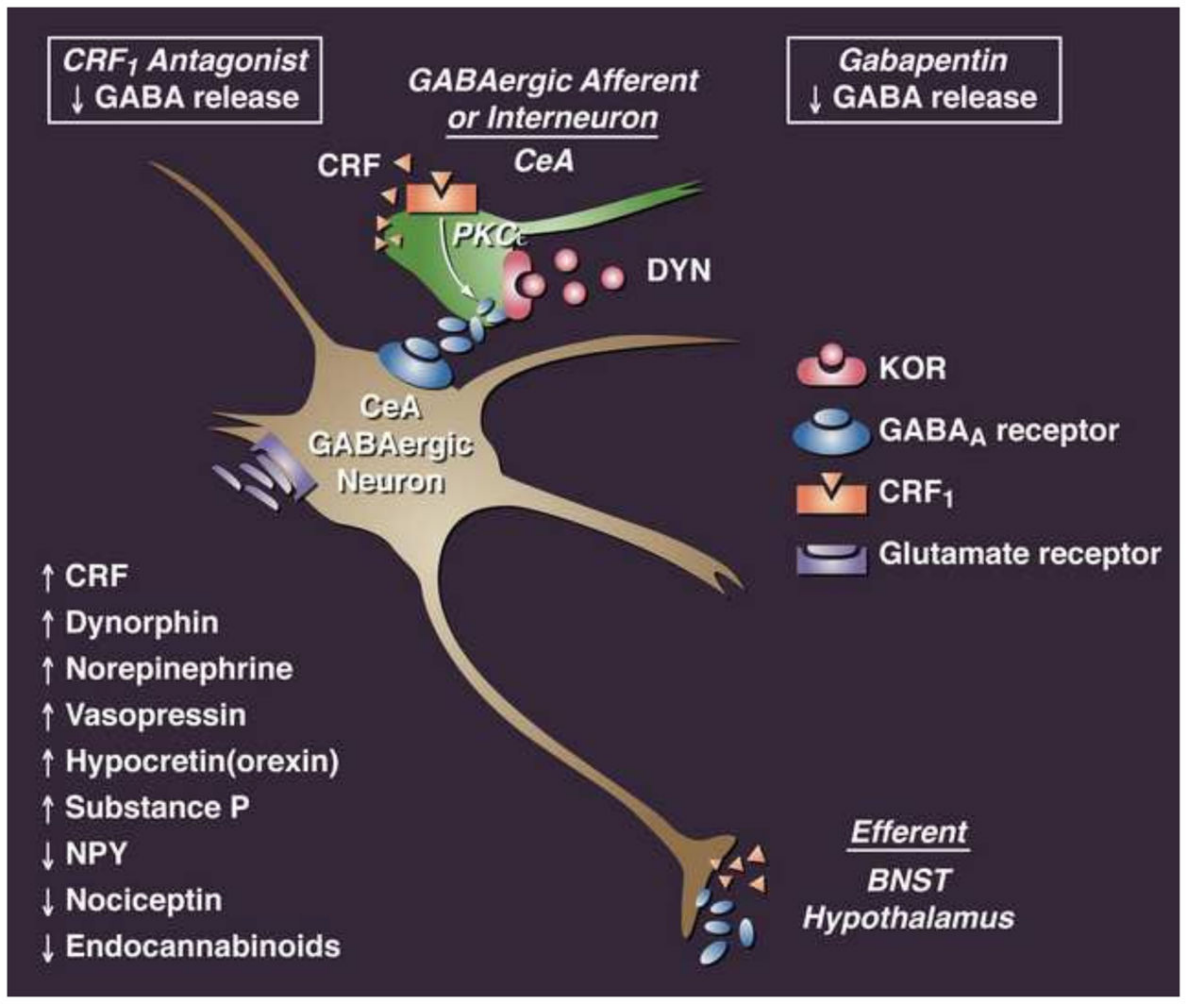
Cellular neuroadaptive mechanisms in the central nucleus of the amygdala in drug addiction. Simplified schematic of rodent central nucleus of the amygdala circuitry and hypothetical sites of ethanol and CRF action on GABAergic synapses. Most neurons in the CeA are GABAergic inhibitory projection neurons or interneurons that contain CRF or other neuropeptides as cotransmitters. (Upper synapse) Ethanol may enhance the release of GABA (filled ellipsoids) from GABAergic afferents or interneurons either via release from the same terminal as CRF (gray triangles), which then acts on CRF_1_ receptors on the terminal to elicit (black arrow) release of more GABA via a PKCε-mediated mechanism, or direct activation of CRF_1_ receptors to elicit the release of more GABA (Bajo et al., 2008). CRF_1_ antagonists and the drug gabapentin decrease presynaptic GABA release in dependent animals (Roberto et al., 2008, 2010). κ-Opioid antagonists have similar effects as CRF_1_ antagonists in rats that present an escalation in cocaine intake (Kallupi et al., 2013). Thus, CRF, dynorphin, and ethanol augment the inhibition of CeA projection interneurons (co-containing CRF, opioids, or NPY), leading to the excitation of downstream (e.g., BNST) neurons through disinhibition. The activation of presynaptic cannabinoid CB_1_ or NPY receptors (data not shown) may reduce GABA release onto CeA inhibitory projection neurons, increasing their excitability and release of GABA onto downstream targets, such as in the BNST. CRF, corticotropin-releasing factor; GABA, γ-aminobutyric acid; CeA, central nucleus of the amygdala; DYN, dynorphi; KOR, κ opioid receptor; NPY, neuropeptide Y; BNST, bed nucleus of the stria terminalis.

The ability of CRF antagonists to block the anxiogenic-like and aversive-like motivational effects of drug withdrawal predicted motivational effects of CRF antagonists in animal models of extended access to drugs. CRF antagonists selectively blocked the increased self-administration of drugs associated with extended access to intravenous self-administration of cocaine (Specio et al., 2008), nicotine (George et al., 2007), and heroin (Greenwell et al., 2009). For example, systemic administration of a CRF_1_ antagonist blocked the increased self-administration of nicotine associated with withdrawal in extended-access (23 h) animals (George et al., 2007). CRF antagonists also blocked the increased self-administration of ethanol in dependent rats during both acute withdrawal and protracted abstinence (Funk et al., 2007). For example, exposure to repeated cycles of chronic ethanol vapor produced substantial increases in ethanol intake in rats during both acute withdrawal and protracted abstinence (2 weeks post-acute withdrawal; O’Dell et al., 2004; Rimondini et al., 2002). Intracerebroventricular administration of a CRF_1_/CRF_2_ antagonist blocked the dependence-induced increase in ethanol self-administration during both acute withdrawal and protracted abstinence (Valdez et al., 2002). Systemic injections of small-molecule CRF_1_ antagonists also blocked the increased ethanol intake associated with acute withdrawal (Funk et al., 2007) and protracted abstinence (Gehlert et al., 2007). When administered directly into the CeA, a CRF_1_/CRF_2_ antagonist blocked ethanol self-administration in ethanol-dependent rats (Funk et al., 2006). These effects appear to be mediated by the actions of CRF on GABAergic interneurons within the CeA, and a CRF antagonist administered chronically during the development of dependence blocked the development of compulsive-like responding for ethanol (Roberto et al., 2010; Fig. 2). The data suggest that CRF itself increases GABA release in the CeA, but a CRF_1_ antagonist blocked the increase in GABA release observed in ethanol-dependent rats, suggesting that the activation of GABAergic interneurons in the central nucleus of the amygdala may reflect neuroadaptations therein that are driven by CRF (Fig. 2). Altogether, these results suggest that CRF in the basal forebrain may also play an important role in the development of the aversive motivational effects that drive the increased drug seeking associated with cocaine, heroin, nicotine, and alcohol dependence.

### 8.2. Dynorphin, compulsive-like drug seeking, and the extended amygdala

As noted above, the excessive release of dopamine and opioid peptides produces subsequent activation of dynorphin systems, which has been hypothesized to feed back to decrease dopamine release and also contribute to the dysphoric syndrome associated with cocaine dependence (Nestler, 2004). Dynorphins produce aversive dysphoric-like effects in animals and humans and have been hypothesized to mediate negative emotional states (Shippenberg et al., 2007; Wee and Koob, 2010; Mucha and Herz, 1985; Pfeiffer et al., 1986).

Enhanced dynorphin action is also hypothesized to mediate the depression-like, aversive responses to stress and dysphoric-like responses during withdrawal from drugs of abuse (Chartoff et al., 2012; Schindler et al., 2010; Land et al., 2009; McLaughlin et al., 2003; Redila and Chavkin, 2008; Land et al., 2008; McLaughlin et al., 2006; Knoll et al., 2007; Mague et al., 2003). For example, pretreatment with a κ-opioid receptor antagonist blocked stress-induced analgesia and stress-induced immobility (McLaughlin et al., 2003), decreased anxiety-like behavior in the elevated plus maze and open field, decreased conditioned fear in fear-potentiated startle (Knoll et al., 2007), and blocked depressive-like behavior induced by cocaine withdrawal (Chartoff et al., 2012).

Recent evidence suggests that the dynorphin-κ opioid system also mediates compulsive-like drug responding (methamphetamine, heroin, and alcohol) with extended access and dependence. κ-Opioid receptor antagonism with a small-molecule κ antagonist selectively blocked responding on a progressive-ratio schedule for cocaine in rats with extended access (Wee et al., 2009). Even more compelling is that excessive, compulsive-like drug and alcohol self-administration can also be blocked by κ antagonists (Walker et al., 2010; Wee et al., 2009; Schlosburg et al., 2013) and may be mediated by the shell of the nucleus accumbens (Nealey et al., 2011; Schlosburg et al., 2013).

### 8.3. Other pro-stress systems, compulsive-like drug seeking, and the extended amygdala

**Figure 3:**
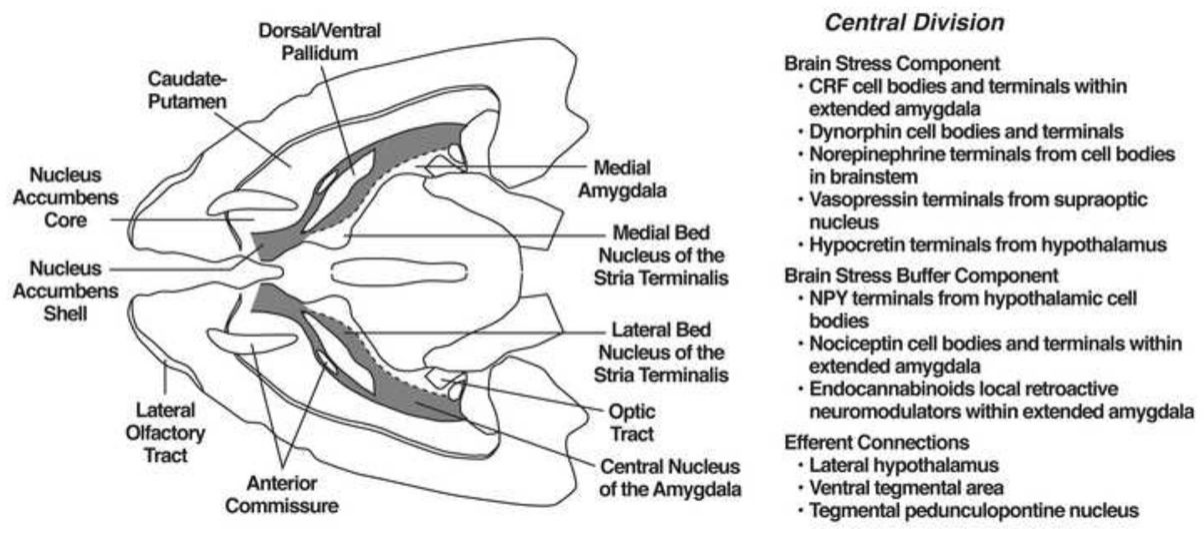
The extended amygdala and its afferent and major efferent connections and modulation via brain arousal-stress systems. Horizontal section through a rat brain depicting the extended amygdala and its afferent and major efferent connections and modulation via brain arousal-stress systems. (Left) Central division of the extended amygdala with the central nucleus of the amygdala and lateral bed nucleus of the stria terminalis and a transition area in the shell of the nucleus accumbens highlighted. (Right) Depiction of the hypothesized interaction of the brain stress systems and brain stress buffer systems and the extended amygdala. Notice that most of the brain stress or brain stress buffer systems are either local circuits or derived from hypothalamic or brainstem discrete groups of cell bodies. Modified from Heimer and Alheid, 1991.

A symphony of pro-stress, pro-dysphoria-inducing brain neurotransmitter systems converge on the extended amygdala. In addition to CRF and dynorphin, there is evidence that norepinephrine, vasopressin, substance P, and hypocretin (orexin) may all contribute to negative emotional states of drug withdrawal, particularly alcohol withdrawal (Koob, 2008). For example using the compulsive-like alcohol seeking model of excessive drinking during withdrawal in dependent rats, a norepinephrine β-adrenoceptor antagonist and a vasopressin 1b antagonist, block excessive drinking (Walker et al., 2008; Gilpin and Koob, 2010; Edwards et al., 2011). A substance P antagonist and hypocretin-2 antagonist blocked compulsive heroin intake (Barbier et al., 2013; Schmeichel et al., 2013). A likely target for these actions is the extended amygdala, suggesting that multiple distributed systems from the brainstem and hypothalamus converge in the extended amygdala to contribute to negative emotional states (Fig. 3). Intriguingly, while activation of this pro-stress, pro-negative emotional state system is multi-determined and comprises the neurochemical bases for hedonic opponent processes described above, there is a multi-determined buffer that may help return the organism to homeostasis if activated.

## 9. Neuropeptide Y, nociceptin, and endocannabinoids: emotional buffer systems

### 9.1. Neuropeptide Y

Neuropeptide Y is a neuropeptide with dramatic anxiolytic-like properties localized to multiple brain regions but heavily innervating the amygdala. It is hypothesized to have effects opposite to CRF in the negative motivational state of withdrawal from drugs of abuse. As such, increases in NPY function may act in opposition to the actions of increases in CRF (Heilig and Koob, 2007). Significant evidence suggests that the activation of NPY in the central nucleus of the amygdala can block the motivational aspects of dependence associated with chronic ethanol administration. Neuropeptide Y administered intracerebroventricularly blocked the increased drug intake associated with ethanol dependence (Thorsell et al., 2005a, b). Neuropeptide Y also decreased excessive alcohol intake in alcohol-preferring rats (Gilpin et al., 2003). Injections of NPY directly into the central nucleus of the amygdala (Gilpin et al., 2008) and viral vector-enhanced expression of NPY in the central nucleus of the amygdala also blocked the increased drug intake associated with ethanol dependence (Thorsell et al., 2007). At the cellular level, NPY, like CRF_1_ antagonists, blocks the increase in GABA release in the central nucleus of the amygdala produced by ethanol (see Fig. 2) and also when administered chronically blocks the transition to excessive drinking with the development of dependence (Gilpin et al., 2011). Again, the activity of GABAergic interneurons in the CeA may reflect the tone of the CeA in balancing brain stress activation via CRF or dynorphin *vs*. brain stress buffer activation via NPY (see Fig. 2). The role of NPY in the actions of other drugs of abuse is limited, particularly with regard to dependence and compulsive drug seeking. Neuropeptide Y_5_ receptor knockout mice have a blunted response to the rewarding effects of cocaine (Sorensen et al., 2009, 2012), and NPY knockout mice show hypersensitivity to cocaine self-administration (Sorenson and Woldbye, 2012). Neuropeptide Y itself injected intracerebroventricularly facilitated heroin and cocaine self-administration and induced reinstatement of heroin seeking in rats with limited access to heroin (Maric et al., 2008, 2009). An NPY Y_2_ antagonist, possibly acting presynaptically to release NPY, blocked social anxiety associated with nicotine withdrawal (Aydin et al., 2011), and NPY injected intracerebroventricularly blocked the somatic signs but not reward deficits associated with nicotine withdrawal (Rylkova et al., 2008). However, the role of NPY in compulsive drug seeking with extended access remains to be studied. The hypothesis here would be that NPY is a buffer or homeostatic response to between-system neuroadaptations that can return the brain emotional systems to homeostasis (Valdez and Koob, 2004; Heilig and Koob 2007). Again, NPY does not act in isolation to reduce stress during alcohol withdrawal. There is evidence that two other neurotransmitter systems, nociceptin and endocannabinoids, may have effects to buffer the activation of brain stress systems.

### 9.2. Nociceptin

Nociceptin (also known as orphanin FQ) is a 17-amino-acid polypeptide that is the endogenous ligand for the nociceptin opioid (NOP) receptor (formerly referred to as opioid receptor-like-1; Meunier et al., 1995; Reinscheid et al., 1995). The neuroanatomical distribution of nociception containing neurons is high in the extended amygdala, cortex, and midbrain. Nociceptin generally attenuates stress-like responses and has a broad anxiolytic-like profile in animals (Ciccocioppo et al., 2003; Martin-Fardon et al., 2010). Nociceptin and synthetic NOP receptor agonists also blocked alcohol consumption in a genetically selected line of rats that is known to be hypersensitive to stressors, decreased reinstatement of drug-seeking behavior (Economidou et al., 2008), and had effects on GABA synaptic activity in the central nucleus of the amygdala, similar to NPY.

### 9.3. Endocannabinoids

Substantial evidence implicates endocannabinoids in the regulation of affective states. Biphasic effects of cannabinoid agonists on anxiety-like behavior have been observed in rats and mice (Viveros et al., 2005), but the effects of endocannabinoids are more consistent. The elevation of interstitial endocannabinoid levels through the inhibition of endocannabinoid clearance mechanisms produces anxiolytic-like effects in various animal models of anxiety, particularly under stressful or aversive conditions, and a reduction of CB_1_ receptor signaling produces anxiogenic-like behavioral effects (Serano and Parsons, 2011). Additionally, the disruption of CB_1_ receptor signaling impairs the extinction of aversive memories, which may have similarities to deficits associated with stress pathophysiology, such as posttraumatic stress disorder (Serano and Parsons, 2011). Several studies indicate that endocannabinoid production is increased in response to stress (Patel et al., 2005). Equally compelling for the current thesis, there is some evidence that drug-seeking behavior can be blocked by endocannabinoid clearance inhibition (Scherma et al., 2008; Adamczyk et al., 2009; Forget et al., 2009). Thus, dysregulated endocannabinoid function may also contribute to the negative affective disturbances associated with drug dependence and protracted withdrawal, which can again be part of the establishment of negative emotional states that drive drug seeking in dependence. As with the other stress buffers discussed above, one could speculate that endocannabinoids play a protective role in preventing drug dependence by buffering the stress activation associated with withdrawal.

## 10. Allostatic Brain Reward and Stress System Changes in Addiction: A Neurocircuitry Perspective

Thus, as dependence and withdrawal develop, incentive salience/reward systems are compromised, and brain anti-reward systems, such as CRF and dynorphin, are recruited in the extended amygdala. We hypothesize that these brain stress neurotransmitters that are known to be activated during the development of excessive drug taking comprise a between-system opponent process, and this activation is manifest when the drug in removed, producing anxiety, hyperkatifeia, and irritability symptoms associated with acute and protracted abstinence. In addition, there is evidence of CRF immunoreactivity in the ventral tegmental area, and a CRF_1_ receptor antagonist injected directly into the ventral tegmental area blocked the social stress-induced escalation of cocaine self-administration (Boyson et al., 2011). Altogether, these observations suggest between-system/within-system neuroadaptations that were originally hypothesized for dynorphin by Carlezon and Nestler (Carlezon et al., 1998), in which the activation of CREB by excessive dopamine and opioid peptide receptor activation in the nucleus accumbens triggers the induction of dynorphin to feed back to suppress dopamine release. Thus, we argue that anti-reward circuits are recruited as between-system neuroadaptations (Koob and Bloom, 1988) during the development of addiction and produce aversive or stress-like states (Nestler, 2001; Koob, 2003; Aston-Jones et al., 1999) via two mechanisms: direct activation of stress-like, fear-like states in the extended amygdala (CRF) and indirect activation of a depression-like state by suppressing dopamine (dynorphin and possibly CRF).

A critical problem in drug addiction is chronic relapse, in which addicted individuals return to compulsive drug taking long after acute withdrawal. This corresponds to the *preoccupation/anticipation* stage of the addiction cycle outlined above. Koob and Le Moal also hypothesized that the dysregulations that comprise the “dark side” of drug addiction persist during protracted abstinence to set the tone for vulnerability to “craving” by activating drug-, cue-, and stress-induced reinstatement neurocircuits that are now driven by a reorganized and possibly hypofunctioning prefrontal system (Koob and Le Moal, 1997, 2001).

The hypothesized allostatic, dysregulated reward and sensitized stress state produces the motivational symptoms of acute withdrawal and protracted abstinence and provides the basis by which drug priming, drug cues, and acute stressors acquire even more power to elicit drug-seeking behavior (Vendruscolo et al., 2012). Thus, the combination of decreases in reward system function and recruitment of anti-reward systems provides a powerful source of negative reinforcement that contributes to compulsive drug-seeking behavior and addiction. A compelling argument can be made that the neuroplasticity that charges the CRF stress system may indeed begin much earlier that previously thought via stress actions in the PFC.

The overall conceptual theme argued here is that drug addiction represents an excessive and prolonged engagement of homeostatic brain regulatory mechanisms that regulate the response of the body to rewards and stressors. The dysregulation of the incentive salience systems may begin with the first administration of drug (Ungless et al., 2001), and the dysregulation of the stress axis may begin with the binge and subsequent acute withdrawal, triggering a cascade of changes, from activation of the HPA axis to activation of CRF in the prefrontal cortex to activation of CRF in the extended amygdala to activation of dynorphin in the ventral striatum. This cascade of overactivation of the stress axis represents more than simply a transient homeostatic dysregulation; it also represents the dynamic homeostatic dysregulation termed *allostasis* (see above).

Repeated challenges, such as with drugs of abuse, lead to attempts of the brain stress systems at the molecular, cellular, and neurocircuitry level to maintain stability but at a cost. For the drug addiction framework elaborated here, the residual decrease in the brain reward systems and activation of the brain stress systems to produce the consequent negative emotional state is termed an *allostatic state*. This state represents a combination of recruitment of anti-reward systems and consequent chronic decreased function of reward circuits, both of which lead to the compulsive drug seeking and loss of control over intake. Where the ventral striatum and extended amygdala project to convey emotional valence, how frontal cortex dysregulations in the cognitive domain linked to impairments in executive function contribute to the dysregulation of the extended amygdala, and how individuals differ at the molecular-genetic level of analysis to convey loading on these circuits remain challenges for future research.

## 11. Hyperkatifeia Revisited: Another Window on the Neurocircuitry of Emotion

The argument that opponent processes play a role in the negative emotional state of drug withdrawal has been extended in parallel to the domain of pain with the hypothesis that repeated administration of opioid medications can produce a hyperalgesic state that can ultimately perpetuate or exaggerate the original pain state being treated. Originally, hyperalgesia was described in animal studies (Celerier et al., 2000; Simonnet, 2005), and hyperalgesia after opioid administration was logically postulated to be an acute sign of opioid withdrawal (Angst and Clark, 2006), a hypothesis compatible with the opponent process theory.

However, now opioid-induced hyperalgesia is considered a clinically relevant phenomenon (Chang et al., 2007; Ossipov et al., 2004, Angst and Clark, 2006) and may be a contributor to the rapid rise in prescription opioid abuse. Opioid-induced hyperalgesia can occur when patients received high doses of opioids for surgical procedures or in patients with a history of high-dose opioid treatment or a prior history of addiction (Angst and Clark, 2006). These findings suggest that chronic or high-dose opioid treatment might be an important contributing factor to the perception of pain in the clinical setting. Yet hyperalgesia alone is unlikely to drive the “switch” to addiction in the small percentage of opioid-treated patients whom are vulnerable to addiction.

One hypothesis to explain the vulnerability to addiction in opioid-treated patients is that chronic pain is well known to cause both emotional distress and negative emotional states (Shurman et al., 2010). An allostatic emotional formulation of the concept of opioid-induced hyperalgesia suggests that a potential *escalating* emotional distress (“emotional pain”) can parallel opioid hyperalgesia during opioid withdrawal that may extend far beyond the physical pain that initiated treatment. This emotional distress was defined as hyperkatifeia. *Hyperkatifeia* (derived from the Greek word *katifeia* for dejection, sadness, or negative emotional state) was defined as the increased intensity of negative emotional/motivational symptoms and signs observed during withdrawal from abused drugs. The term “hyperkatifeia” refers to the increases in emotional distress and emotional pain experienced by individuals with substance use disorders during abstinence. Hyperkatifeia was argued to reflect a pathological change in the emotional “set point” of addicted individuals and is analogous to the term *hyperalgesia* (Shurman et al., 2010). Although severe negative emotional states, including athymia, hypohedonia, and anergy, characterize a variety of psychiatric disorders, including major depressive episodes and schizophrenia, hyperkatifeia is hypothesized to represent more elements such as dysphoria, irritability, alexithymia, or simply symptoms often described as ill at ease, uncomfortable within one’s own skin, or simply not hedonically normal, symptoms historically difficult to define. In short, hyperkatifeia was hypothesized to reflect the hypersensitivity of negative emotional states associated with addiction. This parallel between hyperalgesia in the pain domain and hyperkatifeia in the emotional domain not only links negative emotional states with pain and addiction but also provides another window on how negative emotional states can be generated.

## 12. Emotions Revisited: The Addiction Connection

The thesis of the present article is that emotions consist of “feeling” states and classic physiological emotive responses that are interpreted based on the history of the organism and the context. Interwoven conceptually into this framework is that drugs of abuse elicit powerful emotions, ranging from pronounced euphoria to devastating negative emotional states that in the extreme can create a break with homeostasis and thus an allostatic hedonic state that has been considered key to the etiology and maintenance of the pathophysiology of addiction.

Drugs of abuse produce an abnormal activation of incentive salience/reward systems, such as the release of dopamine in the mesocorticolimbic dopamine system and opioid peptides in the extended amygdala that normally play a key role in guiding behavior toward high-value incentives in the environment. Similarly, one could argue that the opioid peptide system drives incentive salience from the negative reinforcement perspective, encoding salience to stimuli that cause pain or distress. However, the overactivation of dopamine and opioid peptide systems can lead to an immediate opponent process in the emotional domain such that neurotransmitter systems are activated that resemble a stress response. Data to date suggest that CRF, dynorphin, norepinephrine, vasopressin, substance P, and hypocretin are all activated by excessive engagement of incentive salience systems and can contribute to and possibly represent different aspects of negative emotional states. Even more intriguingly, dynorphin and possibly CRF can feed back and actively suppress the incentive salience/reward systems. Finally, for every action there is a reaction, and it is becoming clear that other systems, such as NPY, nociceptin, and endocannabinoids (which can be termed stress buffer systems), are engaged in response to the stress transmitter systems to return the brain emotional circuits to homeostasis. This schema has implications for the etiology and treatment of the excessive engagement of brain incentive salience systems that characterize addiction and also has implications for non-drug negative emotional states.

### 12.1. Implications for our understanding of the neurobiology of emotion

Emotions have a clear role in driving and maintaining motivation and thus it was argued from an evolutionary perspective that the activation of an incentive salience/reward system is a limited resource within a closed system (hedonic Calvinism; Koob and Le Moal, 1997). Thus, it follows that negative emotional states serve multiple purposes. They presumably provide an alarm system for external stressors and threats to physiological, environmental, and social homeostasis; they also serve as an alarm system for excessive engagement in the positive emotional states associated with high incentive salience/reward activity. The argument then is that the brain has specific neurochemical neurocircuitry coded for the hedonic extremes of pleasant and unpleasant emotions that need to be considered in the context of the framework that emotions involve the specific brain regions for interpreting different emotive physiological expression responses.

However, much remains to be understood. What is the neurocircuitry relationship between the brain incentive salience/reward systems (positive emotional systems) and brain stress systems (negative emotional systems) and the neurocircuitry of the six types of emotions outlined above? Are certain brain stress or brain buffer systems disengaged more for a given specific emotion? What are the neurocircuitry connections between the extended amygdala, argued to be a key integrator of the brains stress systems, with the brain incentive salience/reward systems and emotive physiological expression? Presumably, there are key connections with the hypothalamus, periaqueductal gray, and other brainstem systems. How do different brain stress and brain buffer systems interact? In acute dysphoric states, are all the systems engaged? What about in chronic severe major depressive disorder? Much remains to be elucidated, but the present treatise is that the brain stress systems and brain stress buffer systems play key roles in emotional regulation. The impetus for this body of work has been understanding the neurobiology of a severe emotional pathophysiology, addiction, but the implications go far beyond addiction to insights into why human beings not only feel good but also feel bad.
